# Antimicrobial peptide moricin induces ROS mediated caspase-dependent apoptosis in human triple-negative breast cancer via suppression of notch pathway

**DOI:** 10.1186/s12935-023-02958-y

**Published:** 2023-06-21

**Authors:** Imran Ahmad, Saurabh Pal, Ranjana Singh, Khursheed Ahmad, Nilanjan Dey, Aditi Srivastava, Rumana Ahmad, Muath Suliman, Mohammad Y. Alshahrani, Md. Abul Barkat, Sahabjada Siddiqui

**Affiliations:** 1 Department of Biochemistry, King George’s Medical University, Lucknow, 226003 India; 2 Department of Biotechnology, Era’s Lucknow Medical College & Hospital, Era University, Lucknow, 226003 India; 3 Department of Chemistry, BITS- Pilani Hyderabad Campus, Hyderabad, 500078 Telangana India; 4 Department of Biochemistry, Era’s Lucknow Medical College & Hospital, Era University, Lucknow, 226003 India; 5 Department of Clinical Laboratory Sciences, College of Applied Medical Sciences, King Khalid University, Abha, Saudi Arabia; 6 Department of Pharmaceutics, College of Pharmacy, University of Hafr Al-Batin, Al Jamiah, Hafr Al Batin, 39524 Saudi Arabia

**Keywords:** Moricin, Anticancer peptide, Triple negative breast cancer, Notch1, Apoptosis

## Abstract

**Background:**

Breast cancer is the world’s most prevalent cancer among women. Microorganisms have been the richest source of antibiotics as well as anticancer drugs. Moricin peptides have shown antibacterial properties; however, the anticancer potential and mechanistic insights into moricin peptide-induced cancer cell death have not yet been explored.

**Methods:**

An investigation through in silico analysis, analytical methods (Reverse Phase-High Performance Liquid Chromatography (RP-HPLC), mass spectroscopy (MS), circular dichroism (CD), and in vitro studies, has been carried out to delineate the mechanism(s) of moricin-induced cancer cell death. An *in-silico* analysis was performed to predict the anticancer potential of moricin in cancer cells using Anti CP and ACP servers based on a support vector machine (SVM). Molecular docking was performed to predict the binding interaction between moricin and peptide-related cancer signaling pathway(s) through the HawkDOCK web server. Further, in vitro anticancer activity of moricin was performed against MDA-MB-231 cells.

**Results:**

In silico observation revealed that moricin is a potential anticancer peptide, and protein–protein docking showed a strong binding interaction between moricin and signaling proteins. CD showed a predominant helical structure of moricin, and the MS result determined the observed molecular weight of moricin is 4544 Da**. **An in vitro study showed that moricin exposure to MDA-MB-231 cells caused dose dependent inhibition of cell viability with a high generation of reactive oxygen species (ROS). Molecular study revealed that moricin exposure caused downregulation in the expression of Notch-1, NF-ƙB and Bcl2 proteins while upregulating p53, Bax, caspase 3, and caspase 9, which results in caspase-dependent cell death in MDA-MB-231 cells.

**Conclusions:**

In conclusion, this study reveals the anticancer potential and underlying mechanism of moricin peptide-induced cell death in triple negative cancer cells, which could be used in the development of an anticancer drug.

**Supplementary Information:**

The online version contains supplementary material available at 10.1186/s12935-023-02958-y.

## Background

Breast cancer is a multifaceted and widespread disease that imposes a massive strain on the world population due to very high mortality rates [1]. In 2020, there were more than 2.3 million new cases of breast cancer and 685,000 fatalities; and by 2040, it is expected that this number will rise to more than 3 million new cases and 1 million fatalities annually due to population growth and ageing alone [2]. Brest cancer is tragically one of the most common and the primary causes of cancer death in women [3, 4]. Both inherited and non-hereditary factors that influence the onset and course of breast cancer have been identified through epidemiologic investigations. A small percentage of 5 to 10% of breast cancers are hereditary while 90 to 95% of breast cancer are caused by environmental factors [5].Some of the crucial aspects are aging (at early puberty, menopausal as well as first pregnancy) [6], lifestyle, tobacco and alcohol consumption [7], laziness or lack of physical activities, and taking contraceptive pills [8]. Triple-negative breast cancers are notable among breast cancer cases because they lack hormone receptors. These malignancies can be distinguished by their extreme aggressiveness [9]. Like other malignancies, it also has the distinctive ability to avoid apoptosis while progressing toward immortality, which contributes to drug responsiveness [10]. There are two principal apoptosis pathways: extrinsic and mitochondrial (intrinsic). Numerous gene families control these pathways, which ultimately results in cell phagocytosis by neighbouring cells [11]. Although conventional chemotherapies are concerned with the ability to induce apoptosis, many of them are continuously confronted with resistance as well as worrisome adverse effects [12, 13]. Despite improvements in lowering the risk of developing breast cancer, triple-negative breast cancer still has a dismal prognosis [14]. This frenzy fuelled the hunt for reasonably safe natural options to alleviate the clinical implications of hormone-negative breast cancer.

Anticancer Peptides (ACPs) are potent, minute substances that can kill cancer cells by disrupting the mitochondria or by using a membranolytic mechanism [15]. The net negative charge of the cancer membrane is a key factor in peptide selectivity and toxicity when compared to non-cancerous eukaryotic membranes [16]. These peptides can enter malignant cell membranes, compromising membrane integrity, due to their amphiphilicity levels and hydrophobic arc size. For instance, it has been shown that fish-derived Pleurocidin-like Peptides (NRCs) destroy breast cancer cells by disrupting membranes, but only slightly affecting human fibroblasts and human mammary epithelial cells [17]. The delivery of cancer-specific medications may be made possible by these cell-penetrating peptides. Buforin IIb, a non-specific anticancer peptide that penetrates cells, was modified to become more cancer-specific while having little effect on healthy cells [18]. It was discovered that this cancer-specific peptide derivative functioned well in introducing apoptosis-induced antibodies into cancer cells. Notably, the SA12 peptide triggered the apoptosis in breast cancer cells by mitochondrial signalling [19]. Bioinformatics tools for the prediction and design of anticancer peptides (ACPs) were created using the experimentally validated data of physicochemical parameters of peptides. Anticancer peptides predictions servers, AntiCP, and ACP develop support vector machine models (SVM) based prediction from amino acid composition and the presence of an apoptotic domain [20]. In this regard, these prediction tools will aid high-throughput screening for anticancer peptides from complex peptidomes of a variety of natural products.

In recent times, insect-derived antimicrobial peptides (AMPs) have been shown to have a very wide range of activity, such as antibacterial, antifungal, antiviral, as well as anticancerous [21–25]. Silkworms (*Bombyx mori*) are the most valuable assets for sericulture. Interestingly, to protect themselves from pathogenic diseases, silkworms have AMPs as the main component of the immune system [26]. Upon infection, AMPs are quickly released into the hemolymph system to eliminate the pathogenic organism, via disturbing the cell membrane of invading pathogen. Among AMPs, an antimicrobial peptide, named moricin has been reported to show significant antibacterial activity [27, 28]. Moricin, 42 amino acid long, cationic peptide, an α-helix with charged amino acids in the N-terminal half at three to four amino acid residues intervals [26]. Moricin is encoded by various gene families and there are 12 moricin-coding genes are found in the *Bombyx mori* genome, which divided into three subtypes: Bmmor (1 gene), moricin-like A (3 genes), and moricin-like B (8 genes). All these genes produces the mature form of moricin, which has an unaltered amphipathic α–helical N-terminus and a positively charged C-terminus.The absence of this post-translational modification allowed this moricin to be chemically produced [29]. In addition to antimicrobial nature of AMPs, studies have also reported anticancerous activities of some natural AMPs [30, 31]. Recently, Fort et al. have reported the dual role of defensin peptide (TcPaSK) as an antimicrobial and anti-proliferative agent [32]. In the light of earlier studies, we were also interested to assess the anticancerous activity of moricin peptide. Therefore we hypothesized that the anticancerous activity of *B. mori*-derived moricin peptide is yet to be explored. Thus, the present study aimed to predict the putative anti-cancerous properties of moricin peptide by using in silico study and their validation through in vitro model system using MDA-MB-321 cells.

## Methods

### Materials

The supplier of moricin was GL Biochem (Shanghai) Ltd. (Peptide no.147954). The following chemicals were bought from Sigma Aldrich: dimethyl sulfoxide (DMSO), 3-(4, 5-dimethylthiazol-2-yl)- 2,5-diphenyltetrazolium bromide (MTT), lactate dehydrogenase (LDH) (St. Louis, MO, USA). Trypsin-0.25% EDTA solution, Lysotacker red, Mitotracker green, foetal bovine serum (FBS), antibiotic–antimycotic solution, Hoechst 33342 and 2,7-dichlorodihydrofuorescein diacetate (DCFH-DA) were bought from Invitrogen (Waltham, MA, USA). Antibodies against Notch1, NfKB, Bcl-2, Bax, β-actin, Caspase 9, and Caspase 3 were purchased from Abcam (Cambridge, UK). All other substances and reagents were of analytical grade.

### Cell line and culture conditions

The MDA-MB-231 human triple-negative breast cancer cell line was procured from the cell repository centre of the National Centre for Cell Sciences, Pune, India. MDA-MB-231 cells were maintained in DMEM media supplemented with 10% heat-inactivated FBS, 1% penicillin and streptomycin solution and maintained at 37 °C with 5% CO_2_ in an incubator (Thermo Scientific, USA).

### *In-silico* predictions of moricin as anticancer peptide

#### Peptide acquisition

The structure of target peptide moricin was acquired from protein data bank (www.rcsb.org/pdb). Further, cleaning of peptides was performed involving the insertion of missing residues, eliminating water molecules, bound unwanted ligands, and atom uniformity through Accelrys Discovery studio 2017 R2 software.

#### Virtual screening of moricin peptide

Virtual screening of the prospective candidate peptide was predicted using the AntiCP server (http://crdd.osdd.net/raghava/anticp/). The anticancer peptide (AntiCP) server is an open web-based platform to predict the anticancer properties of prospective peptides. A supervised machine learning algorithm ‘Support Vector Machine (SVM)’ aids in the development of models based on the composition of amino acids and binary profile features. iACP (http://lin-group.cn/server/iACP) was used for the validation of SVM scores. Additionally, I-TASSER server (https://zhanggroup.org//I-TASSER/) was utilized to commute secondary structure coordinates of the selected peptides. However, AllerTOP v. 2.0 (https://www.ddg-pharmfac.net/AllerTOP/index.html) and PeptideCutter–Expasy (https://web.expasy.org/peptide_cutter/) bioinformatics tools were implemented to predict the allergenicity and gastrointestinal (GI) digestion resistance of the peptide.

#### Molecular docking

Molecular docking analysis of binding of the peptide with targeted notch signaling pathway molecules including Human DLL4 C2-EGF3 (PDB ID: 5MVX), Human Notch 1 EGF 4–7 (PBD ID: 5FM9), Crystal Structure of Notch 3 Negative Regulatory Region (NRR) (PBD ID: 4ZLP) and Human Jagged-1, domains DSL and EGFs1-3 (PBD ID: 2VJ2) was performed using HawkDOCK web server (http://cadd.zju.edu.cn/hawkdock/). Further the results were validated using another free online platform ‘pyDockWEB’ (https://life.bsc.es/pid/pydockweb).

### Synthesis and characterization of moricin peptide

#### Reverse Phase-high performance liquid chromatography (RP-HPLC)

The purification of peptide was performed in RP-HPLC (Instrument No: 0200194, Lot No: P210205-CL147954) using 4.6 × 250 mm C18 column. Acetonitrile (with 0.1%Trifluoroacetic acid) was used as solvent A, whereas water is used as solvent B (with 0.1%Trifluoroacetic acid). The flow rate was fixed as 1.0 ml/min, while the change in absorbance was monitored at 220 nm. The volume of sample was injected as 10 µL.

#### Mass spectroscopy (MS)

The mass spectrum was recorded using Agilent-6125B instrument. During analysis, the probe bias was fixed at + 4.5 kv, while nebulizer gas flow was kept 1.5 L/min. The H_2_O-acetonitrile (1:1, v/v) was used as solvent with flow rate 0.2 ml/min (detector: 1.5 kv).

#### Circular dichroism (CD)

The CD spectra were recorded on a JASCO instrument, Model J-815-150S. Experiments were performed by purging dry N_2_ gas continuously. Data were collected in a quartz cuvette of 1 mm path length. The spectra were recorded both in water and 30% (v/v) trifluoroethanol-water mixture medium.

#### Cell viability assay

MTT, LDH release, and trypan blue tests were used to evaluate the viability of MDA-MB-231 cells treated with the moricin peptide [33]. Briefly, MDA-MB-321 cells (5 × 10^3^ cells/well) were seeded in 96-well cell culture plates and treated with moricin peptide (0, 0.78, 1.5, 3.1, 6.2, 12.5, 25, 50, 100 µg/ml) and incubated the plate for 24 h in a humified 5% CO_2_ incubator at 37 °C.

#### MTT assay

MTT solution (10.4 mg/ml in PBS) was added to each well after the completion of the treatment time, and the plate was once more incubated in the incubator for the next 4 h at 37 °C. The formazan crystals were then dissolved in 200 μl of DMSO for 30 min, and absorbance was measured at 560 nm (BIORAD-PW41, USA)**.**

#### LHD release assay

The LDH assay kit’s instructions (Sigma Aldrich, MO, USA) were followed to complete the LDH release assay. After 24 h incubation with moricin peptide, 25 μl media was transferred to a new 96- well plate and 50 μl LDH mixture solution added and plate was kept at RT in the dark for 30 min. After dark incubation, 7.5 μl of 1 N HCl were added to the mixed solution and absorbance was measured at 490 nm (BIORAD-PW41, USA).

#### Trypan blue assay

After 24 h incubation, cells were removed using trypsinization. Cells were then centrifuged at 1000 rpm for 5 min, supernatant was discarded carefully and the cell pellet was combined with an equivalent volume of trypan blue (0.4%). Hemocytometer was used for live and dead cell counting.

#### Intracellular reactive oxygen species (ROS) measurement

Fluorescence microscopy imaging and flow cytometry techniques were used to assess intracellular ROS levels using DCFH-DA dye, as reported previously [34]. Briefly, after 24 h, moricin treated and untreated MDA-MB-321 cells was washed with PBS, and then incubated in PBS with 10 µM DCFH-DA dye at 37 °C for 20 min to measure the level of ROS with fluorescent microscope (Zeiss Microsystems, GmBH, Germany) and flow cytometry.

#### Mitochondrial oxygen species (ROS) measurement

Cells (treated or untreated) were incubated with MitoSOX dye (5 µM, Invitrogen) and Hoechst (10 µg/ml) in complete medium and incubated for 30 min at 37 °C. The cells were then washed with 1X PBS for three times and phenol red free complete media was added for imaging under live cell condition. Imaging was done using florescent microscopy (Zeiss Microsystems, GmBH, Germany).

#### Immunofluorescence staining of cells with mitotracker and lysotracker dyes

Cells (treated or untreated) were incubated with Mitotracker dye (75 nM, Invitrogen), Lysotracker dye (100 nM, Invitrogen) and Hoechst (10 µg/ml) in complete medium and incubated for 30 min at 37 °C in 6 well plate. The cells were then washed with 1X PBS for three times and phenol red free complete media was added for imaging under live cell condition. Imaging was done using florescent microscopy (Zeiss Microsystems, GmBH, Germany).

#### Reduced glutathione (GSH) detection

Intracellular GSH levels were estimated using monochlorobimane (100 µM) dye by fluorescence microscopy (Zeiss Microsystems, GmBH, Germany) imaging as reported previously [35]. Additionally, 10% trichloroacetic acid (TCA) was used to examine the glutathione levels within cells. Protein was isolated from moricin treated and untreated MDA-MB-231 cells and precipitated with TCA. TCA-precipitated proteins were briefly vortexed, incubated for 10 min on ice, and then centrifuged at 10,000 rpm for 10 min at 4 °C. Supernatant was taken and mixed with KPE buffer at a 1:1 ratio. O-phthalaldehyde (1 mg/ml) was then taken in equal amount to the diluted samples to prepared reaction mixture and incubated at RT in dark for 10 min. After incubation, the reaction mixture was transported to a multiwall plate reader (BMG FLUOstar Omega) for fluorescence measurement at 355 nm (excitation) and 420 nm (emission). The GSH standard curve was used to determine the GSH level. GSH concentrations are displayed as nmol/g protein [36].

#### Thiobarbituric acid reactive substances (TBARS) assay

Moricin-treated and untreated MDA-MB-231 cells were assessed by the TBARS assay described previously [37]. After 24 h of moricin exposure, the MDA-MB-321cells were homogenized in ice-cold PBS, and cells were lysed through freezing and thawing thrice. TCA (28% w/v in HCl 0.25 M), thiobarbituric acid (1% in acetic acid 0.25 N), and butylhydroxytoluene (125 mM in ethanol) were then taken to mix the homogenate and then the mixture was heated at 95 °C for 15 min and placed in an ice bath. Subsequently, the precipitate was removed by centrifugation at 10,000 g for 15 min at 4 °C, and the absorbance of the supernatant was determined at 535 nm (BIORAD-PW41, USA). TBARS levels were calculated using 1,1,3,3-tetramethoxypropane as the standard.

#### Cell migration assay

Migration assays were performed in 24-well plate with 8-μm pore-sized chamber inserts (Corning Inc., Acton, MA, USA) as described previously [38]. Approximately 4 × 10^4^ cells/well were resuspended in 200 μl of serum-free medium with or without moricin and were then seeded in the upper chambers. In addition, 700 μl of medium supplemented with 10% FBS was added to the lower chambers. The cells were incubated at 37 °C and 5% CO_2_ for 24 and 48 h. After that, cells were fixed with 100% methanol for 20 min and stained with Trypan blue for 30 min. Non-migrating cells on the upper side of the filter were removed with cotton swabs. Migration was quantified by counting the number of cells under an inverted phase contrast microscope.

#### Bromodeoxyuridine (BrdU) assay

The BrdU assay was done with the BrdU kit (Thermo Scientific, USA) as per the manufacturer’s protocol. Imaging was done using fluorescence microscopy (Zeiss Microsystems, GmBH, Germany) imaging.

#### Western blot analysis

Western blotting was performed as per previously established standard procedure [39]. Briefly, MDA-MB-231 cells at a density of 1 × 10^6^ in a T-25 cm^2^ flask were treated with moricin peptide at two effective doses 6.25 and 12.5 µg/mL. Using ice-cold RIPA lysis solution with protease inhibitor cocktail (Thermo Fisher Scientific, USA) the cells were scraped to create the total cell lysates. The total cell lysate was then centrifuged at 13,000 × g for 20 min and the protein concentration in the supernatant was determined with a BCA protein assay kit (Thermo Fisher Scientific, USA) as per manual instruction. An equal quantity of protein samples (30 μg each) were used in SDS-PAGE. Resolved proteins in SDS-PAGE were then transferred onto a PVDF membrane. Filters were then blocked with blocking buffer containing 5% BSA in Tris Buffer Saline Tween 20 (TBST) solution, pH 7.4 under constant agitation for 1 h at 4 °C. Membranes were then incubated overnight at 4^◦^C with primary antibodies of Nocth1 (1:1000), NF-kB (1:1000), p53 (1:1000), Bcl-2 (1:1000), Bax (1:1000), Caspase 3 (1;1000), Caspase 9 (1:1000) and β-actin (1:1000) as per the dilutions suggested by the manufacturer. The membrane was then washed thrice with TBST and incubated with horseradish peroxidase (HRP)-conjugated secondary antibodies (1:5000) for 1 h at RT with gentle shaking. After washing, bands were visualized by ECL Western Blotting Substrate Kit (Thermo Fischer Scientific, USA) according to the manufacturer’s instructions. The relative abundance of each band was quantified using Image Studio, lite version 5.2 software (LI-COR), and normalized to β-actin as a loading control.

### Statistical analysis

Three biological replicates (nine technical replicates) were performed for all experiments. Data were analysed using Graph-Pad Prism 7.05 Software (GraphPad Software, Inc., La Jolla, CA, USA). The results are presented as the mean ± SD, and statistical significance was determined using one-way ANOVA, and two way ANOVA followed by Dunnett’s multiple comparisons test. A statistically significant value was considered at p < 0.05.

## Results

### Physicochemical, allergenicity, digestion resistance and anticancer assessment of moricin peptide

The antibacterial peptide ‘moricin’ consists of 42 amino acid sequences. The physicochemical properties reveal that moricin is a highly basic entity, and greater the basicity, the more anti-microbial effects it possesses **(**Table 1**).** The electrostatic association(s) between positively charged moricin peptide and negatively charged bacterial membrane is mediated with an isoelectric point (pI) calculated to be at 11.3. The instability index value was 8.32. Furthermore, the peptide did not possess any toxic, allergenic and digestion resistance responses as predicted by the in silico study. Anticancer prediction servers (AntiCP and iACP) with SVM score 0.75 and 0.99 reveal their potent anticancer property prediction (Table 2). Additionally, the SVM score of 0.16 has been over served for the cell-penetrating property of the peptide (Table 2). The iDNA-Prot server revealed that the peptide can interacts with the nucleic acids and the interacting amino acids predicted were Tyr27, Glu30, Val31, Asp33, and Phe34 (Table 3). The C-score value ranges from 0–1 predicting for ligand binding where, a higher score indicates a more reliable prediction. The secondary structures of the peptide were predicted using NPS@: SOPMA secondary structure prediction tool (https://npsa-prabi.ibcp.fr/NPSA/npsa_sopma.html) (Table 2).

**Table 1:** Prediction of physicochemical, allergic, and digestive properties of moricin peptide

S.No	Properties	Observation
1	Allergenicity	Probable non-allergen
2	GI digestion resistance	No
3	Hydrophobicity	− 0.18 (47.62%)
4	Steric hindrance	0.62
5	Sidebulk	0.62
6	Hydropathicity	− 0.21
7	Amphipathicity	0.94
8	Hydrophilicity	0.25
9	Net hydrogen	0.88
10	Charge	10.50
11	Isoelectric point (pI)	11.37
12	Molecular weight (MW)	4544.16
13	GRAVY	− 0.21
14	Instability index	8.32 (Stable)

**Table 2:** Potential anticancer properties prediction of moricin peptide

SVM score^a^			AntiCP prediction^b^	iACP prediction^c^	iDNAprot^d^	CPP^e^	Predicted secondary Structure^f^		
**AntiCP**	**iACP**	**CPP**	Anticancer	Anticancer	DNA binding protein	Penetrate cell membrane	**Helix**	**Coil**	**Strand**
0.75	0.993363	0.16					57.14%	26.19%	11.90%

^**a**^SVM score of the peptide given by the AntiCP server, iACP server and ToxinPred server respectively

^**b,c**^Anticancer property of peptide predicted by AntiCP and iACP server respectively

^**d**^Predicting DNA-binding proteins by iDNA-Prot server

^**e**^Prediction of Cell Penetrating Peptides by CPPpred server

^**f**^Secondary structure predicted by SOPMA

**Table 3:**
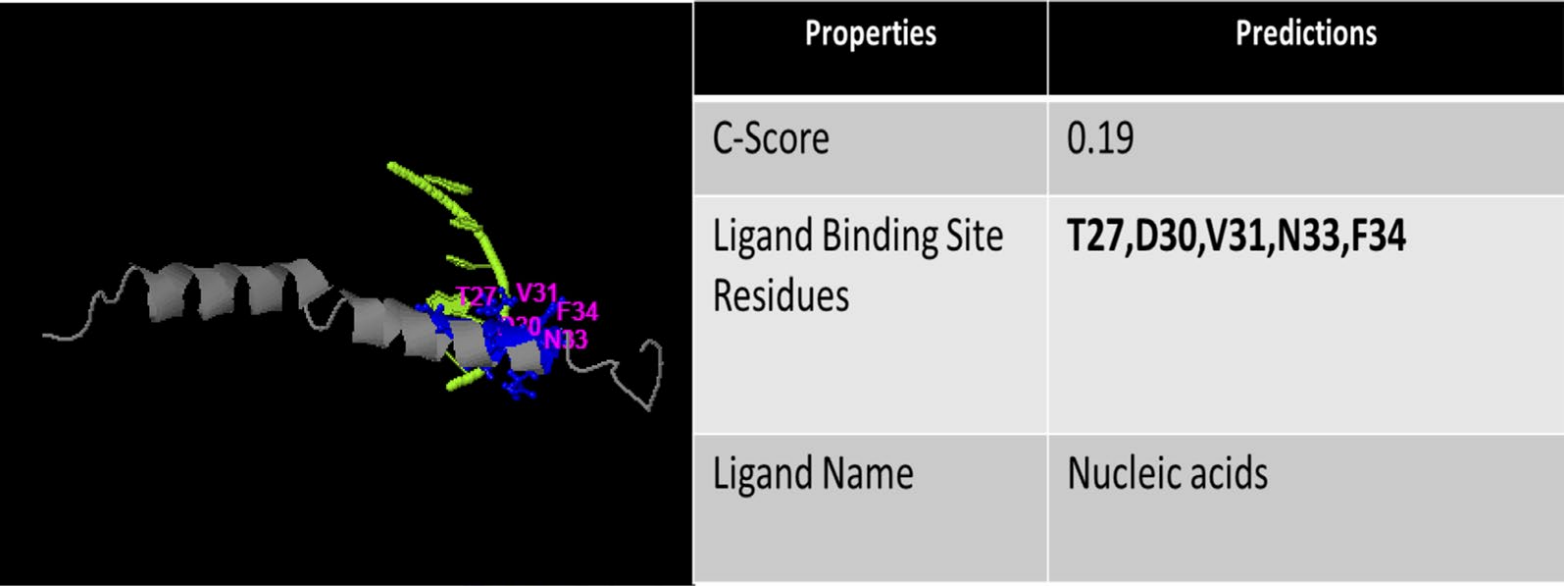
Peptide Nucleic Acid Binding Sites Predicted by I-TASSER server

In left panel, represents peptide moricin binds the nucleic acid, displaying the interacting amino acid residues. Right panel represents the C-score which ranges [0–1], where a higher score indicates a more reliable prediction. Predicted Result: Protein moricin may be DNA binding protein

### Docking analysis

Protein-peptide interactions where moricin was docked with targeted proteins in the notch signaling pathway namely human DLL4, crystal structure notch 1, notch 3, and jagged-1 proteins. By analyzing the individual docked models generated shows that maximum binding of moricin peptide was observed with notch 3 NRR with a binding free energy (BFE) of − 72.13 kcal/mol and the interacting amino acids are depicted in table (Table 4). As evident from results, the binding affinity of moricin with targeted proteins decreased in the order: notch 3 (BFE: − 72.13 kcal/mol) > DLL4 (BFE: − 59.79 kcal/mol) > TACE (BFE: − 51.14 kcal/mol) > notch 1 (BFE: − 48.92 kcal/mol) > jagged-1 (BFE: − 41.49 kcal/mol).

**Table 4:** Summary of moricin peptide docked with selected target proteins using hawkdock server

S. No	Target protein	Protein-peptide docked structure	BFE of complex (kcal/mol)	VDW	ELE	GB	SA	IAA
	Crystal Structure of Notch3 Negative Regulatory Region (PBD ID: 4ZLP)		− 72.13	− 96.16	− 3214.77	3251.9	− 13.1	**Protein:** LeuA:196, LeuA:194, TrpA:111, AspA:331, AspA:262, AspA:244, GluA:119, GlnA:102, HieA:75, AspA:454 **Peptide:**ArgB:20, IleB:24, LysB:9, ThrB:10, LysB:13, LysB:6, IleB:3, LysB:2, PheB:32, LeuB:35
	Human DLL4 C2-EGF3 (PDB ID: 5MVX)		− 59.79	− 92.35	− 950.43	995.32	− 12.33	**Protein:** AspA:247, GluA:226, GluA:248, AspA:256, HieA:265, AsnA:225, ValA:293, TyrA:282, GlyA:199, LeuA:297 **Peptide:** IleB:5, IleB:24, *ValB:31*, ArgB:20, ThrB:27, IleB:8, LysB:2, AsnB:23, *PheB:34*, ProB:4
	Crystal structure of TACE (PDB ID: 2FV5)		− 51.14	− 45.87	− 1397.16	1399.41	− 7.52	**Protein:** GlnA:471, AspA:298, GluA:464, AspA:470, GluA:295, GluA:469, AspA:310, AspA:458, AspA:485, ProA:330 **Peptide:** ArgB:20, *PheB:34*, LysB:9, IleB:24, LysB:38, LysB:17, LysB:2, IleB:5, PheB:32, LysB:13
	Human Notch-1, EGF 4–7 (PBD ID: 5FM9)		− 48.92	− 53.35	− 1267.44	1280.09	− 8.21	**Protein:**AspA:119, GluA:115, GluA:116, GluA:154, AspA:134, ProA:122, AspA:120, IleA:118, GlnA:112, TyrA:151 **Peptide:**ILeB:24, ArgB:20, *ValB:31*, LysB:13, ThrB:27, PheB:32, LysB:6, LysB:17, AsnB:23, AlaB:28
	Human Jagged-1, domains DSL and EGFs1-3 (PBD ID: 2VJ2)		− 41.49	− 97.94	− 1226.38	1294.61	− 11.78	**Protein:** ValA:86, TrpA:102, AspA:108, HieA:87, AspA:110, AspA:168, GluA:148, ValA:236, TyrA:142, IleA:299 **Peptide:** PheB:32, IleB:24, ArgB:20, LysB:17, LysB:2, IleB:5, LysB:13, IleB:8, IleB:3, ProB:4

*BFE* Binding Free Energy, *VDW* Van der Waals force, *ELE* Electrostatics, GB:, SB:, *IAA* Interacting Amino acids

### Moricin is α-helix predominant structure with a MW of 4543.5 Da revealed by CD and LC/MS

**Fig. 1:**
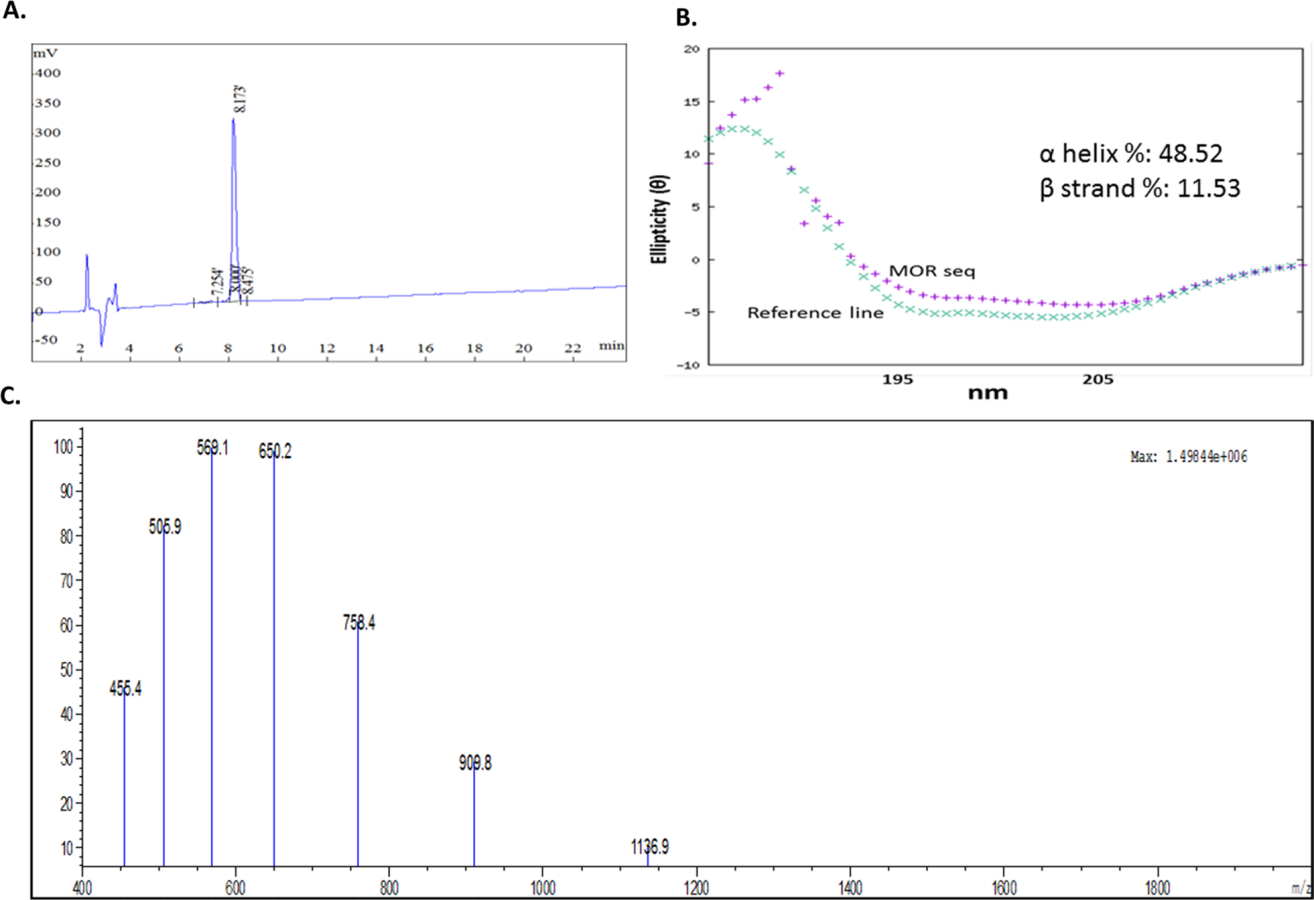
Synthesis and characterization of moricin peptide **A** RP-HPLC chromatogram shows purified moricin peptide **B** The CD spectra of peptide in 30% (v/v) trifluoroethanol-water mixture. **C** Mass spectrum of purified moricin peptide (unmodified C-terminus)

Peptide was analyzed and purified by reversed-phase high-performance liquid chromatography (RP-HPLC) with a linear gradient of water (0.1% TFA). The RP-HPLC chromatogram of purified peptide showed one major peak at approximate retention time 8.1 min (Fig. 1A). Most of the contaminants and unwanted side products were eluted before 4 min. When the amount of organic solvent was very less (< 10%), the specific fraction was collected (peak of interest) and then freeze-dried before used for other applications. The purified peptide was further characterized by mass spectrometry and circular dichroism. The CD spectrum of synthesized moricin peptide showed negative band at ~ 190 nm in water, indicating random coil conformation. However, when analysed in presence of 30% (v/v) trifluoroethanol-water mixture, two distinct minima were observed at 197 and 208 nm region. In addition, a positive band was observed at ~ 182 nm. This indicates α-helix as the predominant secondary structure. A closer look into the position and relative intensity of the bands indicate presence of 44.4% α-helix and 13.5% β-strand structure (Fig. 1B). For further confirmation, the molecular mass of the peptide was measured by ion spray mass spectrometry on a single quadrupole LC/MS spectrometer. The obtained value, 4544.0 ± 0.4 Da, was coincident with the literature-reported value, 4543.5 Da (Fig. 1C) considering that the C terminus of the peptide is unmodified.

### Moricin induces growth inhibition of MDA-MB-231 cells at 6.25 µg/ml concentration

**Fig. 2:**
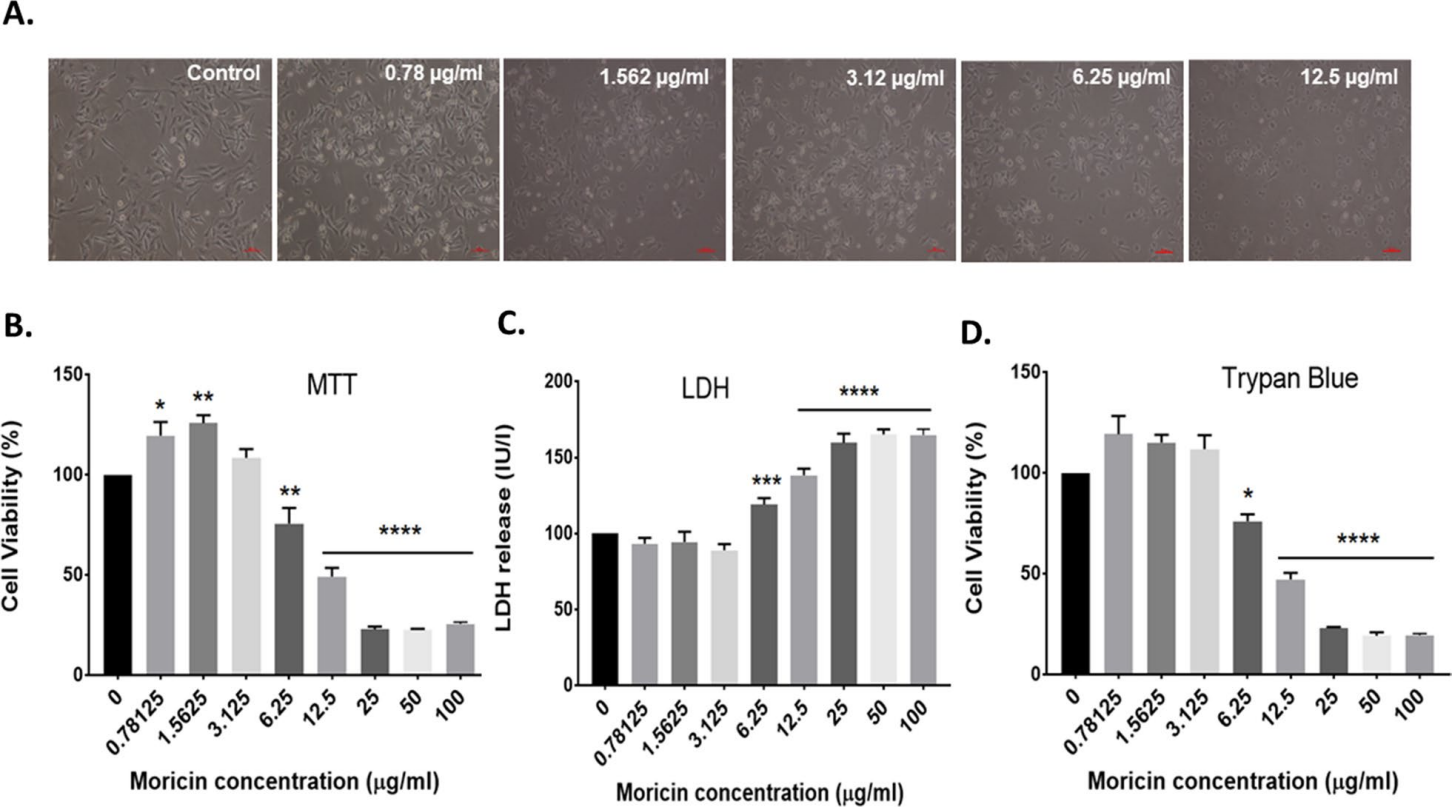
Effect of moricin peptide treatment on cell viability in MDA-MB-231 cells (**A**–**D**) Determination of toxic doses of moricin in MDA-MB-231 cells. Cells were cultured and incubated for 24 h without moricin (untreated) and with different concentrations of moricin (0–100 100 µg/ml). **A** Represents the morphology of moricin treated MDA-MB 231 cells with respect to untreated cells. Images were taken with Zeiss Axio-Observer, Germany at 20X magnification at scale bar 200 µm (**B**, **C**) represents the percent of cell viability and toxicity with respect to untreated cells as measured by MTT, LDH, and trypan blue assay respectively. Bar graphs are expressed as mean ± S.E of three independent experiments. Statistical analysis was performed using one way ANOVA test followed by Dunnett’s post hoc comparison test *p < 0.05, **p < 0.01, and ***p < 0.001 is significant as compared to the untreated cells

To determine the effective dose of moricin peptide on MDA-MB-231 cells, we assessed the moricin peptide-induced cytotoxicity in MDA-MB-231 cells by measuring cell viability. As shown (Fig. 2A, B), cells were treated with various concentrations of moricin peptide (0–100 μg/ml or 0–22 μM) for 24 h. Moricin peptide exposure up to 3.125 μg/ml (0.688 μM) did not cause any significant loss in cell viability. However, the cell viability was found to be decreased significantly at 6.25 (1.37 μM) and 12.5 μg/ml (2.75 μM) concentrations of peptide by 24% and 50%, respectively. While at above concentrations more than 75% decrease in cell viability was observed as compared to untreated cells. Additionally, the cytotoxic effects of moricin peptide were also confirmed by assessing LDH release (Fig. 2C) and trypan blue assay (Fig. 2D) Similar to MTT assay, results of LDH release and trypan blue assays also showed significant decrease in cell viability at 6.25 μg/ml and above concentrations of moricin peptide. Hence, based on these results, we selected 6.25 (1.37 μM) and 12 μg/ml (2.75 μM) concentrations of moricin peptide for our subsequent studies.

### Moricin induces intracellular ROS generations in MDA-MB-231 cells

**Fig. 3:**
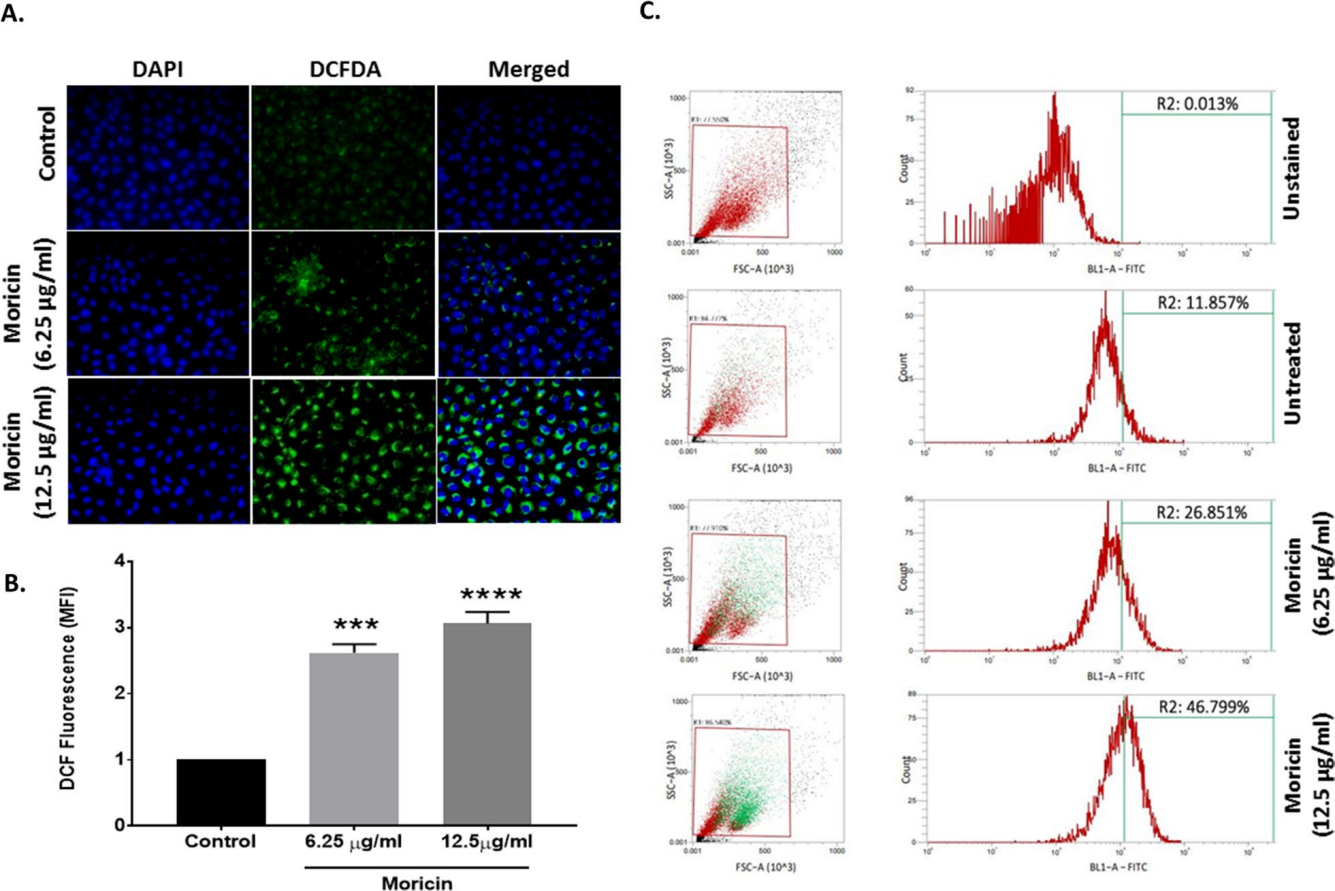
Effect of moricin peptide treatment on intracellular ROS in MDA-MB-231 cells **A** Microscopy analysis were performed in MDA-MB-231 cells to measure intracellular ROS, cells were exposed to 6.25 µg/ml and 12.5 µg/ml concentrations of moricin to measure ROS with H2-DCFDA. Images were taken with florescent microscopy (Zeiss Microsystems, GmBH, Germany) at 20X magnification **B** Represents the mean fluorescence of DCFDA at 30 min. The fluorescence intensity of DCFDA was determined at 515/490 nm. **C** Represents the flow cytometric analysis to measure ROS with H_2_-DCFDA staining. Results are the mean ± S.E from three independent experiments and statistical analysis was determined one-way ANOVA test followed by Dunnett’s post hoc comparison test **p < 0.01, and ***p < 0.001 vs untreated cells

There are several growing evidences that anticancer drugs exposure causes significant alteration in the cellular redox status of cancer cells. Therefore, an effect of moricin peptide on cellular redox in MDA-MB-231 cells was tested. As shown (Fig. 3A, B), moricin exposure causes significant rise of 1.5 and twofold in the levels of intracellular ROS levels at doses 6.25 and 12.5 μg/ml, respectively. Flow cytometry results revealed the intracellular ROS were 11.85% in untreated cells, while 26.85% and 46.80% at 6.25 and 12.5 μg/ml moricin treated cells, respectively (Fig. 3C).

### Moricin induces ROS mediated structural damage to mitochondria and lysosome

**Fig. 4:**
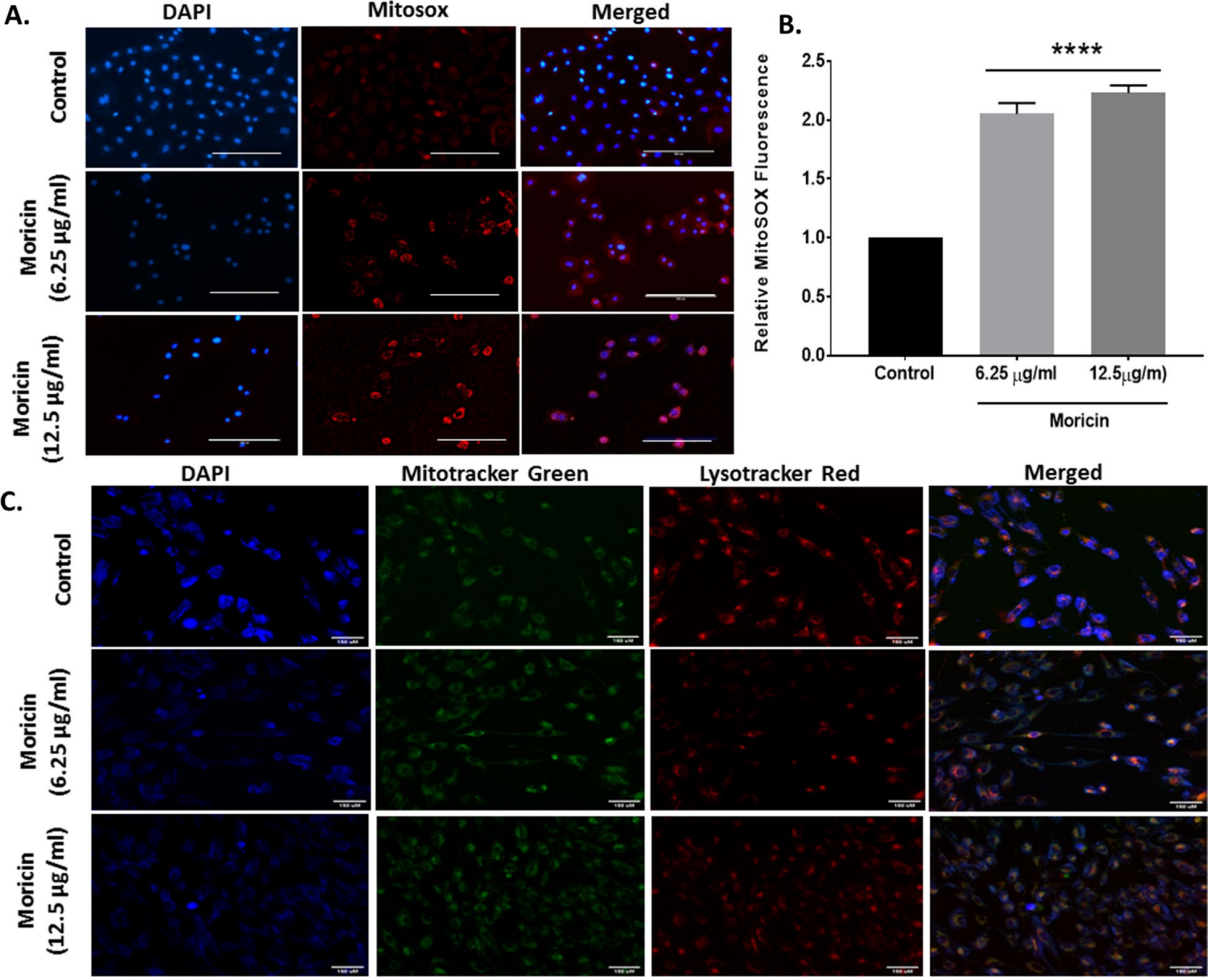
Effect of moricin peptide treatment on Glutathione and TBARS level in MDA-MB-231 cells **A** Microscopy analysis were performed in MDA-MB 231 cells to measure GSH in cells treated with 6.25 µg/ml and 12.5 µg/ml concentrations of moricin using Monochlorobimane (mBCI) staining. Images were taken with florescent microscopy (Zeiss Microsystems, GmBH, Germany) at 20X magnification at scale bar 50 µm **B** Represents the level of GSH (nmol/µg) in MDA-MB-231 cells treated with 6.25 µg/ml and 12.5 µg/ml concentrations of moricin. **C** Represents the level of TBARS (µM) in MDA-MB-231 cells treated with 6.25 µg/ml and 12.5 µg/ml concentrations of moricin. Results are the mean ± S.E from three independent experiments and statistical analysis was determined one-way ANOVA test followed by Dunnett’s post hoc comparison test *p < 0.05, and ***p < 0.001 vs untreated cells

Several studies have reported that more than 90 percent of cellular ROS is generated through mitochondria; therefore the effect of moricin exposure on mitochondrial ROS was tested. Moricin exposure caused significant increase of mitochondrial superoxide anion by 1 and 1.4 fold in 6.25 and 12.5 μg/ml moricin treated cells, respectively as compared to control (Fig. 4A, B). Further, the effects of superoxide anion at lysosome and mitochondrial morphology were also observed using fluorescence microscopy. Significant morphological alteration were observed in the mitochondria and lysosomal structure at 12.5 µg/ml of moricin concentration. However at low doses (6.25 µg/ml), no significant morphological alterations were observed in both mitochondria and lysosome (Fig. 4C).

### Moricin reduces intracellular GSH level and enhances TBARS in MDA-MB-231 cells

**Fig. 5:**
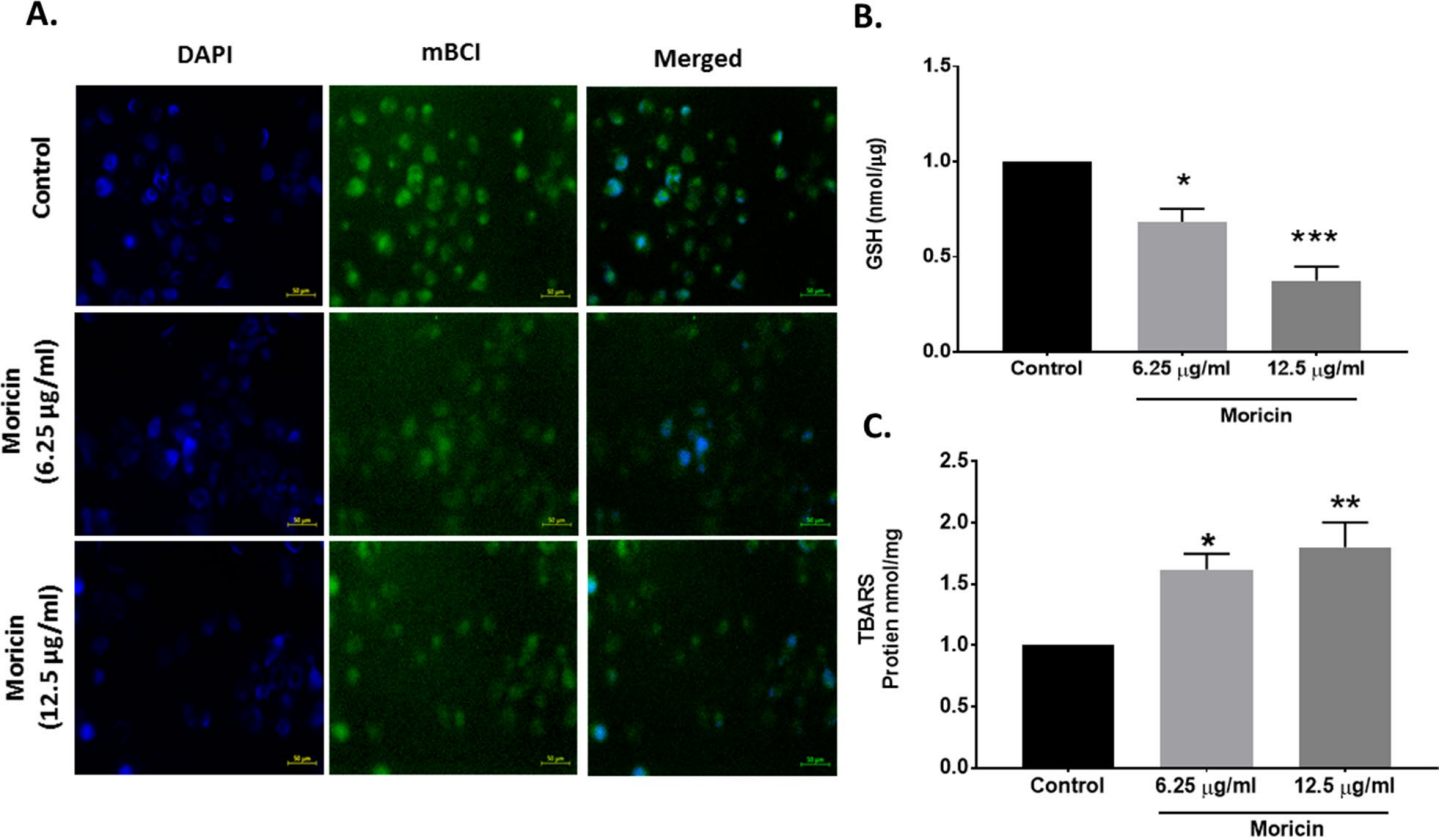
Effect of moricin peptide treatment on structural damages of mitochondria and lysosomes and induced mitochondrial ROS generations in MDA-MB-231 cells **A** Mitochondrial ROS was determined by microscopy with MitoSOX staining of moicin treated and untreated MDA-MB-231 cells treated with 6.25 µg/ml and 12.5 µg/ml moricin peptide. Images were taken withflorescent microscopy (Zeiss Microsystems, GmBH, Germany) at 20X magnification at scale bar 100 µm. **B** Represents level of mitochondrial ROS through relative mitoSOX florescence. **C** Fluorescent images of the MDA-MB-231 cells treated with 6.25 µg/ml and 12.5 µg/ml moricin peptide. Staining was done for the nucleus (blue), mitochondria (green) and lysosome (red) using Hoechst 33,342 (10 µg/ml), Mitotracker Green FM (75 nM), and Lysotracker Red (100 nM). Cell shrinkage and mitochondrial disruption are seen for cells treated with moricin but not in the control cells. Images were taken with florescent microscopy (Zeiss Microsystems, GmBH, Germany) at 20X magnification at scale bar 150 µm Results are the mean ± S.E from three independent experiments and statistical analysis was determined one-way ANOVA test followed by Dunnett’s post hoc comparison test ****p < 0.0001 vs untreated cells

Effect of moricin on oxidative stress induced cellular glutathione and lipid peroxidation was studied. Results showed that moricin exposure leads to the significant decrease in the total intracellular glutathione levels (Fig. 5A, B) whereas, the TBARS assay results showed the significant rise in the level of lipid peroxidation in a dose dependant manner (Fig. 5C**)**.

### Moricin retards proliferation, migration and colony formation rate in MDA-MB-231 cells

**Fig. 6:**
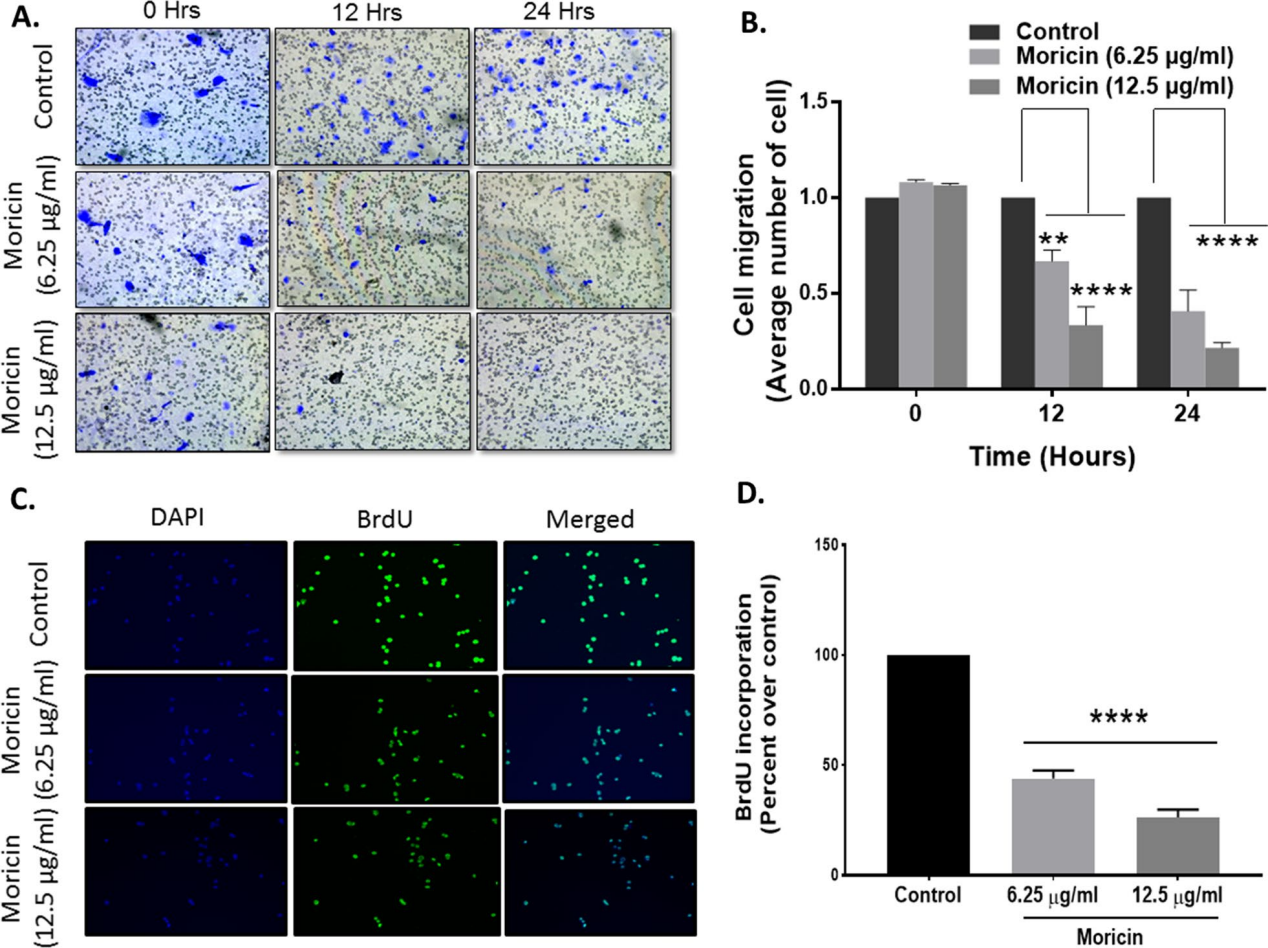
Effect of moricin peptide treatment on cell migration in MDA-MB-231 cells **A** Migration assays showed that MDA-MB-231 cells exposed to 6.25 µg/ml and 12.5 µg/ml moricin peptide had decreased migration and invasion potential compared with control **B** statically results from A. **C** Fluorescent images of the MDA-MB-231 cells treated with 6.25 µg/ml and 12.5 µg/ml moricin peptide and staining with BrdU and **D** BrdU incorporation in MDA-MB-231 cells treated with 6.25 µg/ml and 12.5 µg/ml moricin. The bar graphs are presented as mean ± S.E of three independent experiments and statistical analysis was determined by two-way ANOVA test followed by Dunnett’s post hoc comparison test **p < 0.01, and ****p < 0.0001 vs untreated cells

As shown in Fig. 6A, B, moricin causes a significant decline in the rate of migration after 12 and 24 h exposure. However, 12.5 μg/ml concentration of morcin shows relatively higher anti-migration effects after 24 h. Similarly, BrdU incorporation also showed the significantly decrease in the rate of cell proliferation after moricin exposure and caused 60 and 70% lower cell proliferation at doses 6.25 and 12.5 μg/ml, respectively (Fig. 6C, D). Soft agar assay (Additional file 1: Fig. S1) also showed the significant decreased colony formation in moricin treated cells.

### Moricin induces caspase dependent cell death *vi*a down regulating the Notch-1/NF-ƙB in MDA-MB-231 cells

**Fig. 7:**
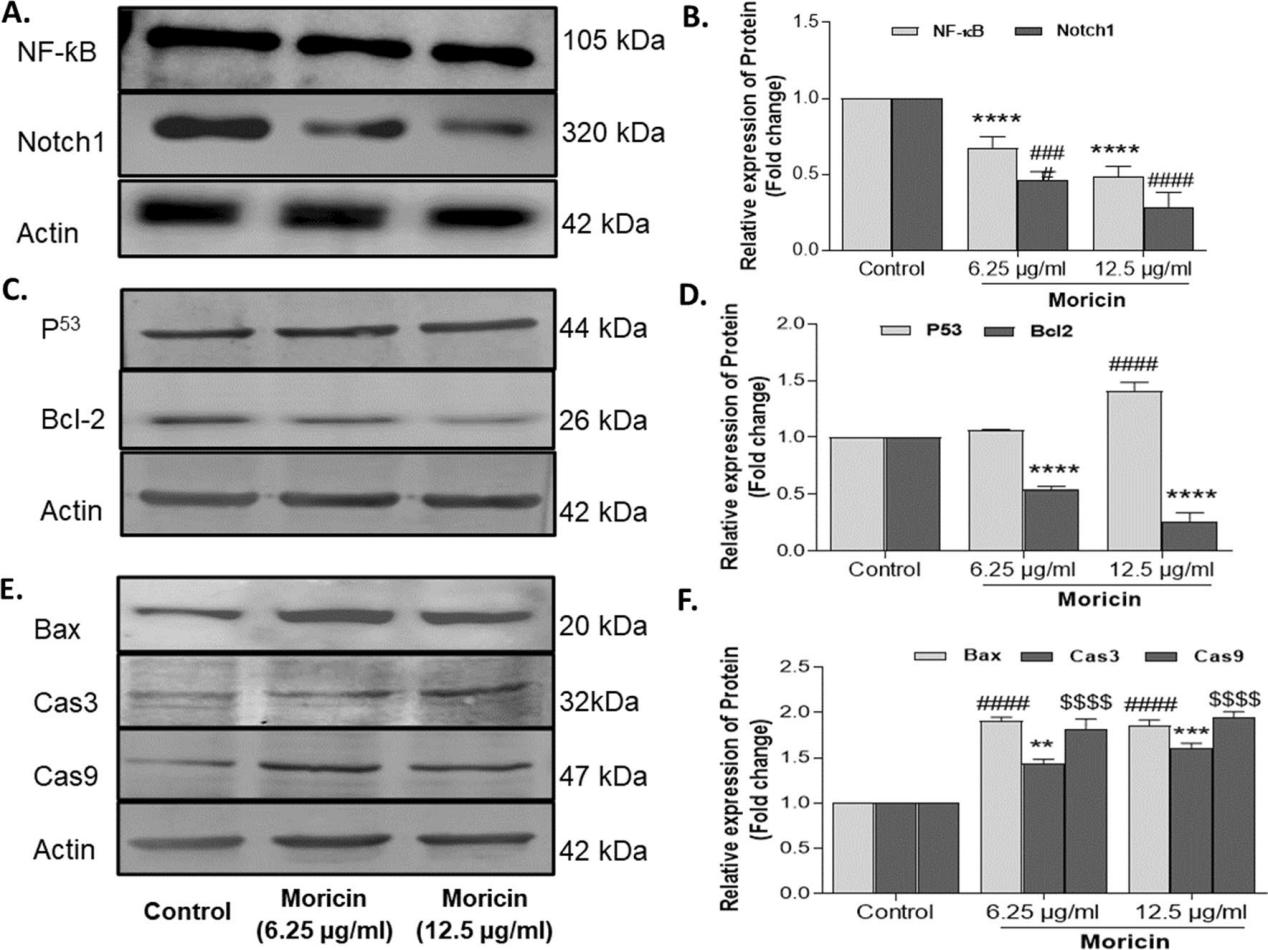
Moricin exposure leads to Notch1/NF-ƙB mediated activation of programme cell death **A** Western blot analysis of NF-ƙB, Notch1, **C** p53, Bcl-2 and **E** Bax, caspase3 and 9 protein expression respectively in moricin exposed MDA-MB-231 cells treated with 6.25 µg/ml and 12.5 µg/ml moricin peptide. **B** Densitometric analysis of NF-ƙB and Notch1 protein bands (fold change) as compared to untreated cells, after normalization with β-actin. **D** Densitometric analysis of p53 and Bcl-2 protein bands (fold change) as compared to untreated cells, after normalization with β-actin. **F** Densitometric analysis of Bax, caspase3 and 9 protein bands (fold change) as compared to untreated cells, after normalization with β-actin. Densitometric analysis of bands was performed using Image studio, lite version 5.2 software (LI-COR) and values are expressed as fold change after normalization with β-actin. The bar graphs are presented as mean ± S.E of three independent experiments and statistical analysis was determined by two-way ANOVA test followed by Dunnett’s post hoc comparison test **p < 0.01, ***p < 0.001 and ****p < 0.0001 vs untreated cells, ####p < 0.0001 vs untreated cells and $$$$p < 0.0001 vs untreated cells

To validate in silico binding affinity and for molecular pathways analysis of morcin induced cell death, immunoblotting was performed in moricin exposed MDA-MB-231 cells. Result showed that level of expression of both Notch-1 and NF-ƙB proteins significantly decreased in moricin exposed cells as compared to untreated cells (Fig. 7A, B**).** The expression level of tumor suppressor protein p53 was significantly increased in moricin treated cells (Fig. 7C, D). Subsequently, the level of protein expression of apoptotic pathways viz*.* pro-apototic protein Bad, initiator caspase-9, and effector caspase-3 were upregulated significantly as compared to control cells **(**Fig. 7E, F). Interestingly, expression level of anti-apoptotic Bcl-2 protein was significantly downregulated (Fig. 7C, D).

## Discussion

In the present study, we have predicted and examined the potential anti-cancer property of moricin peptide obtained from *Bombyx mori***,** utilizing in silico and in vitro model systems. The present in silico findings stipulated that moricin is a cationic peptide with remarkable anticancer property. It can permeate through the cell membrane and, has a peculiar DNA binding sequence of amino acid residues. Molecular docking analysis revealed that moricin peptide has high binding affinity (BFE = − 48.92 kcal/mol) with human Notch-1 receptor protein. Following that, an in vitro study was performed to validate our in silico findings. Our findings demonstrate that exposing MDA-MB-231 cells to moricin peptide causes intracellular oxidative stress and superoxide anion formation in the mitochondria, resulting in morphological alterations in the mitochondria, lysosomes, and nucleus. Furthermore, the molecular study unveiled that moricin peptide may trigger the apoptotic pathway(s) via Notch-1 mediated modulation of NF-kB, Bcl2, and p53 proteins.

**Fig. 8:**
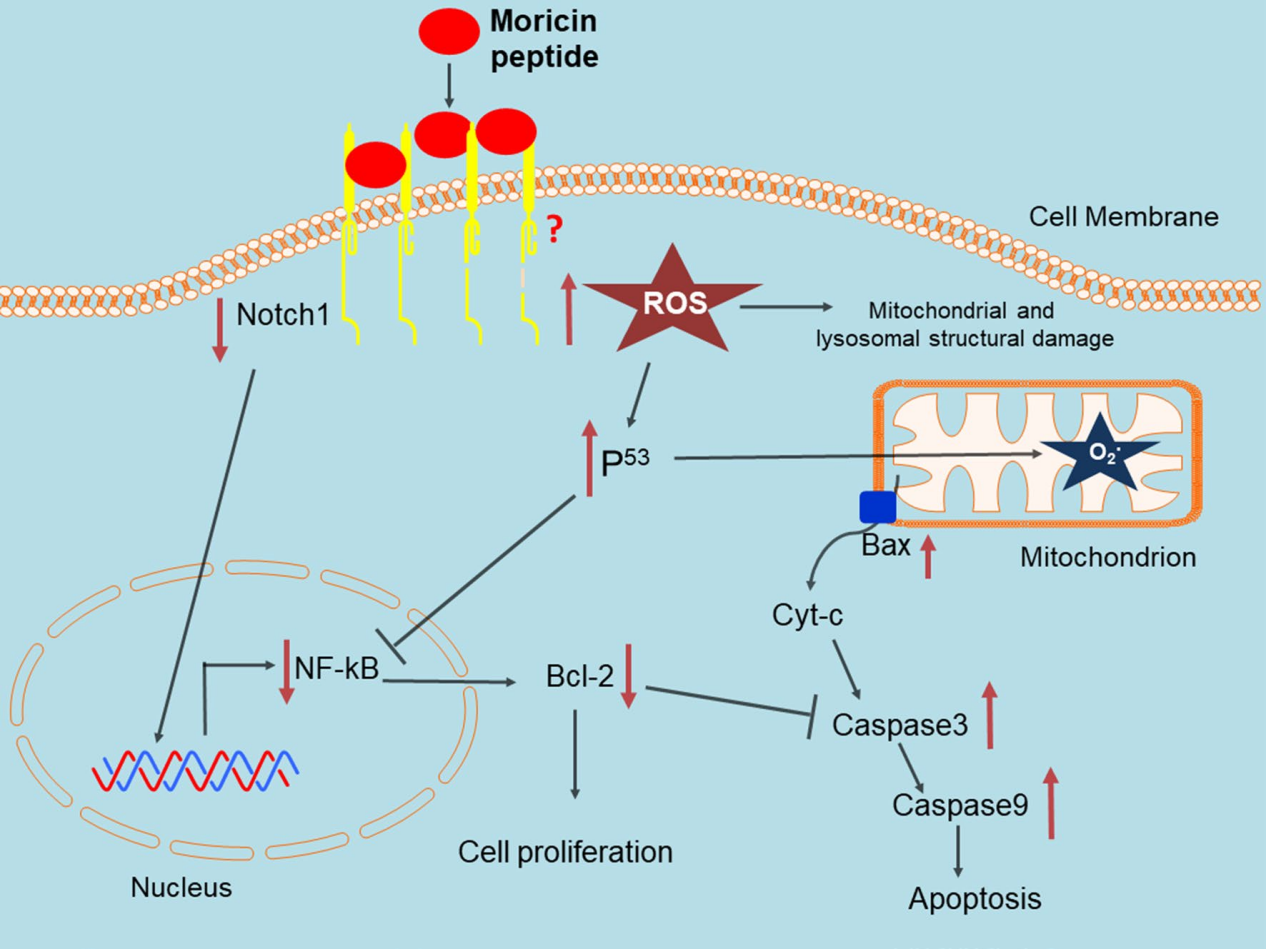
Schematic representation of moricin induced cell death in MDA-MD-321 cells

Research over the last three decades has shown that naturally occurring peptides have antibacterial properties. However, various studies have revealed that these natural peptides may have some additional properties such as immunomodulatory activity, antiviral, antifungal, and anticancer effects [19, 22, 40]. Owing to the multifactorial effects of these natural peptides, we have screened and selected the moricin peptide from *Bombyx mori* (silkworm). This peptide is produced as a component of their innate immune system to defend against the bacterial infection [26]. Thus, the antimicrobial activity of moricin is well documented. The antibacterial peptide moricin consists of a sequence with a highly basic entity, and the greater the basicity, the more anti-microbial effects it possesses [27]. *To the best of our knowledge, no study has investigated the anticancer property of this peptide against cancer including triple-negative breast cancer*. Therefore, to explore the anticancer potential, initially, an in silico study of moricin peptide with targeted proteins was performed. As evident from predictions, AntiCP and iACP servers with SVM scores of 0.75 and 0.99 have predicted their potent anticancer property. Additionally, the SVM score of 0.16 was overserved for the cell-penetrating property of the peptide (Table 2). The iDNA-Prot server has revealed that the peptide interacts with the nucleic acids and the interacting amino acids were Tyr27, Glu30, Val31, Asp33, and Phe34 (Table 3). The secondary structures of the peptide were anticipated to have a higher percentage of α-helices than coil and β-strand. In recent years, an increasing number of α-helical peptides have been discovered as ACPs [41, 42]. Wang et.al., have shown that α-helical peptide L-K6 can enter MCF7 cells through micropinocytosis and inhibit cancer cell death by causing substantial damage to the nucleus [43]. Similarly, Liu has reported that anti-microbial α-helical peptide induces death in cancer cells [44]. Interestingly, our study has also shown the similar anticancer effects of moricin peptide on cancer cells. It might be possible as moricin peptide has the electrostatic association(s) between positively and negatively charged ions with an isoelectric point (pI) calculated to be at 11.37 (Table 1). Since the membranes of bacteria and cancer cells have a similar net negative charge, therefore cationic peptides can kill both bacteria and cancer cells [45]. As evident from the data, the instability index value of 8.32 confirms the stability of the peptide which might be due to the presence of a larger number of α-helices in the structure. Moreover, various other parameters such as cationic hydrophobic and amphiphilicity may also contribute significantly to enhancing the anticancer effect by imparting cytotoxicity to cancer cells [46, 47]. Furthermore, the peptide did not possess any allergenic and digestion resistance responses. Protein-peptide docking was used to investigate the binding affinity of moricin peptide to the cancer cell signalling-associated marker. Among several marker proteins Notch protein family shows strong binding with the peptide. Thus, considering it we further investigated the Protein-peptide based interaction with the other members’ proteins of notch signalling viz. human DLL4, crystal structure notch 1, notch 3, and jagged-1 proteins (Table 4). To validate in silico predictions, we have procured the moricin peptide with its physiochemical characterization to ensure its properties. The purified peptide chromatogram revealed a single main peak (indicating peptide purity) at about a retention time of 8.1 min [48]. Additionally, CD and LC/MS spectrum study reveals that moricin peptide is predominately have α-helical structure with a molecular weight of 4544 Da, (Fig. 1). Our data was also consistent with the literature-reported value of moricin peptide [49, 50]. Next, our in vitro findings reinforced the anticancer properties of the peptides by showing a significant decline in the number of cancer cell viability at relatively low concentrations (1.37 and 2.75 µM), which indicates that moricin can efficiently disrupt the growth and proliferation of cancer cells (Fig. 2). Kuroda et. al. have assess the cytotoxicity of membrane permeable peptide in breast cancer cells at 0.5–1 mM concentrations [51], likewise, Li et.al., have shown that nanoparticle assembled peptides (12–54 µM) have significantly increases cytotoxicity in ovarian and lung cancer cells [52]. It is well known that ROS has been implicated in various cellular processes and metabolic pathways of cancer cells, thus perturbation in cellular thiol levels would directly affect the growth of cancer cells [34, 53]. Interestingly, our results also showed that moricin even at relatively low concentrations (6.25 μg/ml and 12.5 g/ml) causes significantly higher levels of ROS in the cytosol and mitochondria (Figs. 3 and 4A and B). Furthermore, it should be noted that the generation of free reactive superoxide (O2•–) species in mitochondria is well established to disrupt the oxidative phosphorylation at the inner mitochondrial membrane, which eventually culminates in ATP deprivation [34]. Therefore, it might be possible that moricin-induced formation of free reactive superoxide (O2•–) in mitochondria may play a significant role to limit cancer cell growth and proliferation. Nevertheless, these speculations are further confirmed by significant alteration in the morphology of mitochondria (Fig. 4). In continuation, the considerable decrease in the intracellular glutathione levels in moricin-exposed cells demonstrates the mortician's deleterious effects on the cellular redox state in cancer cells (Fig. 5). Moreover, this unusual redox imbalance causes a considerable increase in lipid peroxidation [54], which is supposed to alter the wide variety of lipids present in the membrane of various cell organelles such as mitochondria and lysosomes. As lipids play an essential role in maintaining the structure and integrity of the cell membranes. Various studies have shown that free radicals can directly interfere with chromosomal integrity, thus may disturb the central dogma [55]. Interestingly, our BrdU assay results also show that moricin causes a dramatic reduction in the rate of BrdU incorporation (Fig. 6C, D), which signifies that moricin exposure to cancer cells may disrupt the process of cell replication, and as a consequence, cell proliferation may be diminished (Fig. 6A, B) [56]. Moreover, various studies have reported the critical interplay between Notch1, NF-kB, and p53, which plays a decisive role in determining the proliferative and apoptotic fate of cancer cells [57–59]. For that reason, we were also interested to explore the effect of moricin peptide on the expression of Notch1protein. Intriguingly, our In silico study has revealed the strong binding interaction of moricin peptide to the notch-1 receptor, therefore, in connection with our in silico findings we have speculated that moricin peptide may inhibit the expression of notch-1 protein through the notch-1 receptor. Interestingly, our molecular study has shown that moricin peptide has significantly decreased the expression of Notch-1 protein and its downstream signaling pathway- related protein (i.e. NF-kB) in moricin peptide treated cells. Furthermore, the expression of p53 protein was also found to be markedly upregulated in moricin peptide exposed cells, these findings indicate that a decrease in Notch-1 protein expression is inversely related to the upregulation of p53 protein. Moreover, it is well-established that higher expression of ROS in cancer cells may trigger the higher expression of the p53 protein [60]. It is widely known that p53 acts as a tumor suppressor gene and its higher expression causes the induction of a caspase-dependent cell death pathway in cancer cells [53]. Likewise, our findings have also shown similar results by upregulating the expression of p53, Bax, Caspase 3, and Caspase 9 while downregulating the expression of anti-apoptotic Bcl2 protein (Figs. 7, and 8).

## Conclusions

In brief, our study indicates that treatment with moricin in MDA-MB-231 cells results in ROS-induced, caspase-dependent cell death via down regulation in the expression of Notch-1 and, NF-ƙB and Bcl2 proteins.

## Supplementary Information

**Additional file 1: Figure S1**. Soft agar assay: MDA-MB 321cells were grown in soft agar with and with outmoricin (6.25and12.5μg/ml) for 28days, and the image of colonies were captured by EVOS Core Cell Imaging System with the Scale bar of 100μm.

## Data Availability

All data generated/analysed during this study are included in this article. The datasets used in the study are available from the corresponding authors on reasonable request.

## Abbreviations

RP-HPLC: Reverse Phase-high performance liquid chromatography
MS: Mass spectroscopy
CD: Circular dichroism
ACPs: Anticancer peptides
SVM: Support Vector Machine
NRCs: Pleurocidin-like Peptides
AMPs: Antimicrobial peptides
DMSO: Dimethyl sulfoxide
MTT: 3-(4, 5-Dimethylthiazol-2-yl)- 2,5-diphenyltetrazolium bromide
LDH: Lactate dehydrogenase
TBARS: Thiobarbituric acid reactive substances
BrdU: Bromodeoxyuridine
