# Correction: Antimicrobial peptide moricin induces ROS mediated caspase-dependent apoptosis in human triple-negative breast cancer via suppression of notch pathway

**DOI:** 10.1186/s12935-023-02988-6

**Published:** 2023-07-15

**Authors:** Imran Ahmad, Saurabh Pal, Ranjana Singh, Khursheed Ahmad, Nilanjan Dey, Aditi Srivastava, Rumana Ahmad, Muath Suliman, Mohammad Y. Alshahrani, Md. Abul Barkat, Sahabjada Siddiqui

**Affiliations:** 1 Department of Biochemistry, King George’s Medical University, Lucknow, 226003 India; 2 Department of Biotechnology, Era’s Lucknow Medical College & Hospital, Era University, Lucknow, 226003 India; 3 Department of Chemistry, BITS-Pilani Hyderabad Campus, Hyderabad, 500078 Telangana India; 4 Department of Biochemistry, Era’s Lucknow Medical College & Hospital, Era University, Lucknow, 226003 India; 5 Department of Clinical Laboratory Sciences, College of Applied Medical Sciences, King Khalid University, Abha, Saudi Arabia; 6 Department of Pharmaceutics, College of Pharmacy, University of Hafr Al-Batin, Al Jamiah, Hafr Al Batin, 39524 Saudi Arabia

**Correction: Cancer Cell International (2023) 23:121** 10.1186/s12935-023-02958-y

In this article [1], the figure legends for Figures 4 and 5 was interchanged. The correct Figs. 4 and 5 with legends are given in this erratum.

**Fig. 4:**
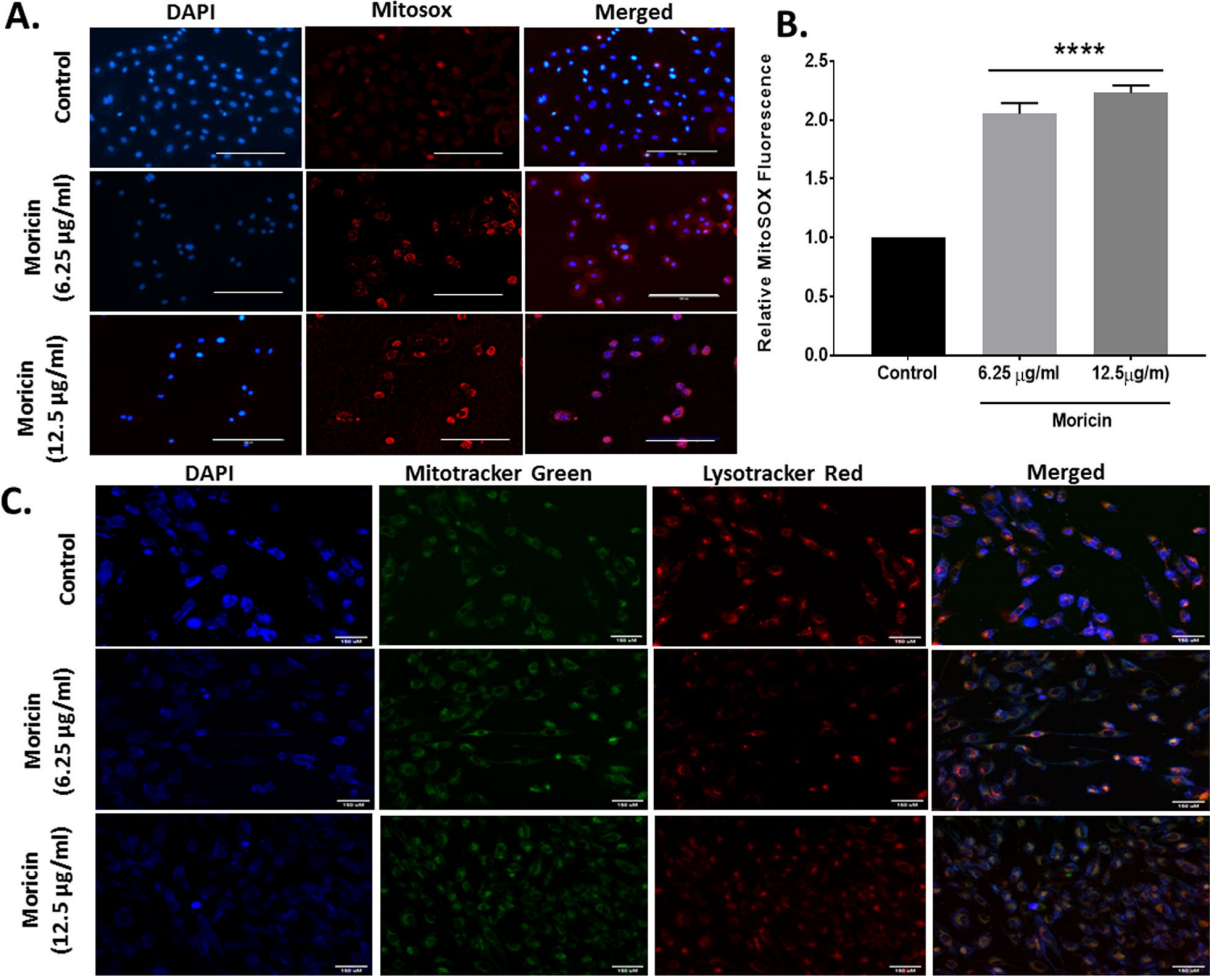
Effect of moricin peptide treatment on structural damages of mitochondria and lysosomes and induced mitochondrial ROS generations in MDA-MB-231 cells. **A** Mitochondrial ROS was determined by microscopy with mitoSOX staining of moricin treated and untreated MDA-MB-231 cells treated with 6.25 µg/ml and 12.5 µg/ml moricin peptide. Images were taken with florescent microscopy (Zeiss Microsystems, GmBH, Germany) at ×20 magnification at scale bar 100 µm. **B** Represents level of mitochondrial ROS through relative mitoSOX florescence. **C** Fluorescent images of the MDA-MB-231 cells treated with 6.25 µg/ml and 12.5 µg/ml moricin peptide. Staining was done for the nucleus (blue), mitochondria (green) and lysosome (red) using Hoechst 33342 (10 µg/ml), Mitotracker Green FM (75 nM), and Lysotracker Red (100 nM). Cell shrinkage and mitochondrial disruption are seen for cells treated with moricin but not in the control cells. Images were taken with florescent microscopy (Zeiss Microsystems, GmBH, Germany) at ×20 magnification at scale bar 150 µm. Results are the mean ± S.E from three independent experiments and statistical analysis was determined one-way ANOVA test followed by Dunnett’s post hoc comparison test ****p < 0.0001 vs untreated cells

**Fig. 5:**
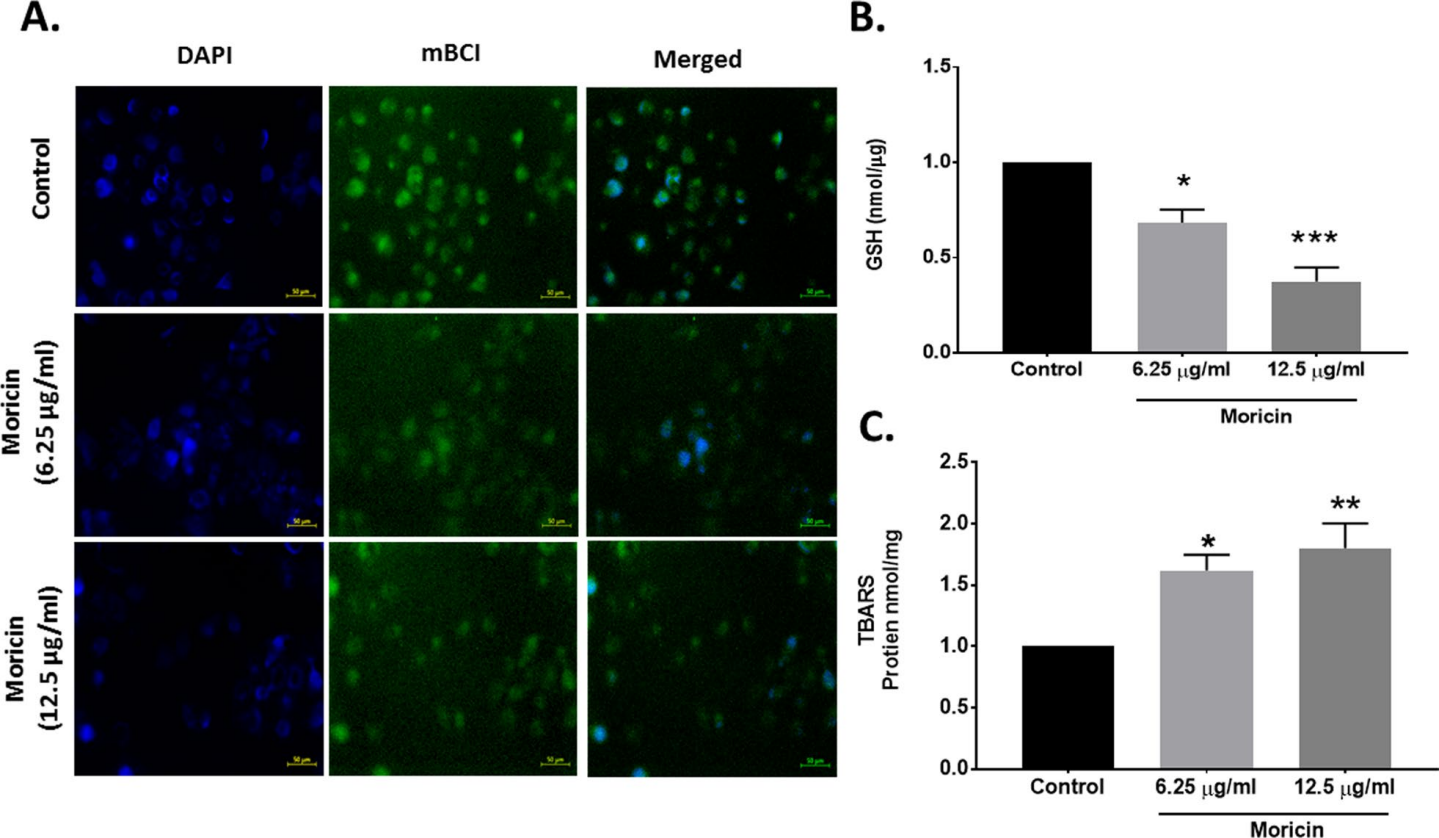
Effect of moricin peptide treatment on Glutathione and TBARS level in MDA-MB-231 cells. **A** Microscopy analysis were performed in MDA-MB 231 cells to measure GSH in cells treated with 6.25 µg/ml and 12.5 µg/ml concentrations of moricin using Monochlorobimane (mBCI) staining. Images were taken with florescent microscopy (Zeiss Microsystems, GmBH, Germany) at ×20 magnification at scale bar 50 µm. **B** Represents the level of GSH (nmol/µg) in MDA-MB-231 cells treated with 6.25 µg/ml and 12.5 µg/ml concentrations of moricin. **C** Represents the level of TBARS (µM) in MDA-MB-231 cells treated with 6.25 µg/ml and 12.5 µg/ml concentrations of moricin. Results are the mean ± S.E from three independent experiments and statistical analysis was determined one-way ANOVA test followed by Dunnett’s post hoc comparison test *p < 0.05, and ***p < 0.001 vs untreated cells
