# Supplementary material for: Antimicrobial peptide moricin induces ROS mediated caspase-dependent apoptosis in human triple-negative breast cancer via suppression of notch pathway
Source: Cancer Cell Int. 2023 Jun 21;23:121. doi: 10.1186/s12935-023-02958-y (PMC10283202; doi:10.1186/s12935-023-02958-y)
Supplement: Supplementary file 1 — Additional file 1: Figure S1. Soft agar assay: MDA-MB 321cells were grown in soft agar with and with outmoricin (6.25and12.5μg/ml) for 28days, and the image of colonies were captured by EVOS Core Cell Imaging System with the Scale bar of 100μm. [file 12935_2023_2958_MOESM1_ESM.pdf]

Additional file

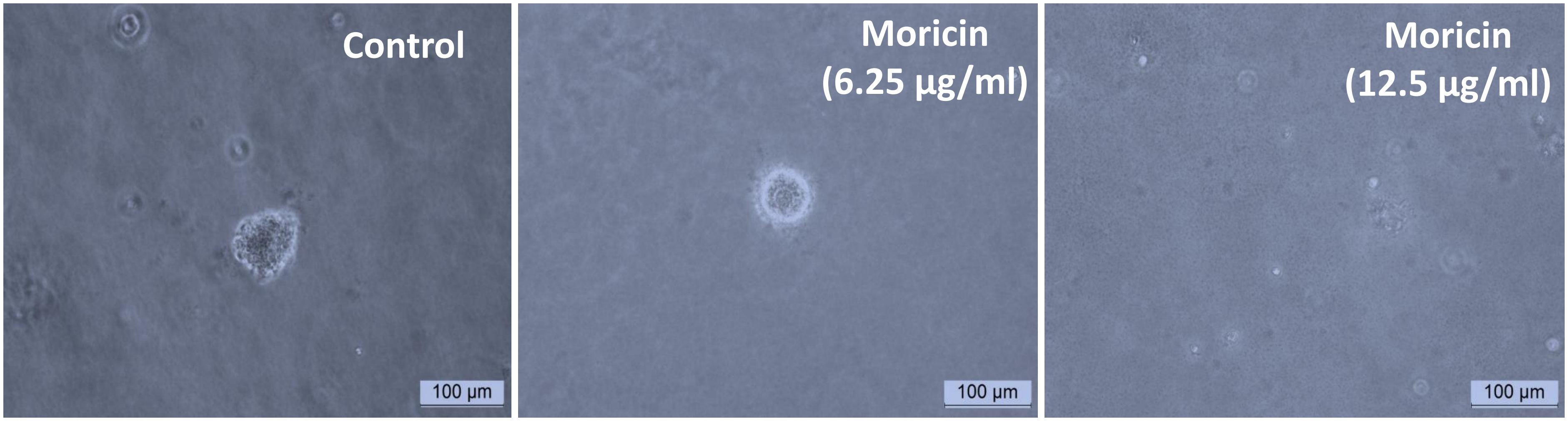

Figure S1: Soft agar assay: MDA-MB-321 cells were grown in soft agar with and without moricin (6.25 and 12.5µg/ml) for 28 days, and the image of colonies were captured by EVOS Core Cell Imaging System with the Scale bar of 100 µm .
